# Nutrients and bioactive compounds content of *Baillonella toxisperma, Trichoscypha abut* and *Pentaclethra macrophylla* from Cameroon

**DOI:** 10.1002/fsn3.217

**Published:** 2015-03-09

**Authors:** Robert Fungo, John Muyonga, Archileo Kaaya, Clement Okia, Juius C Tieguhong, Jojo J Baidu-Forson

**Affiliations:** 1 School of Food Technology, Nutrition & Bio-Engineering, Makerere University, P.O. Box 7062, Kampala, Uganda; 2 Forest Genetic Resources Programme, Bioversity International, Via dei Tre Denari, 472/a 00057, Maccarese, Rome, Italy; 3 World Agroforestry Centre (ICRAF), Uganda Country Office, P.O Box 26416, Kampala, Uganda

**Keywords:** Forest foods, human nutrition, nutrient composition and Congo Basin regionnutrient

## Abstract

*Baillonella toxisperma*, *Pentaclethra macrophylla* and *Trichoscypha abut* are important foods for communities living around forests in Cameroon. Information on the nutritional value and bioactive content of these foods is required to establish their contribution to the nutrition and health of the communities. Samples of the three foods were obtained from four villages in east and three villages in south Cameroon. The foods were analyzed for proximate composition, minerals and bioactive content using standard chemical analysis methods. *T. abut* was found to be an excellent source of bioactive compounds; flavonoids (306 mg/100 g), polyphenols (947 mg/100 g), proanthocyanins (61.2 mg/100 g), vitamin C (80.05 mg/100 g), and total oxalates (0.6 mg/100 g). *P. macrophylla* was found to be a rich source of total fat (38.71%), protein (15.82%) and total fiber (17.10%) and some bioactive compounds; vitamin E (19.4 mg/100 g) and proanthocyanins (65.0 mg/100 g). *B. toxisperma,* was found to have high content of carbohydrates (89.6%), potassium (27.5 mg/100 g) and calcium (37.5 mg/100 g). Flavonoids, polyphenols, vitamins C and E are the main bioactive compounds in these forest foods. The daily consumption of some of these fruits may coffer protection against some ailments and oxidative stress. Approximately 200 g of either *B. toxisperma* or *P. macrophylla,* can supply 100% iron and zinc RDAs for children aged 1–3 years, while 300 g of the two forest foods can supply about 85% iron and zinc RDAs for non-pregnant non-lactating women. The three foods provide 100% daily vitamins C and E requirements for both adults and children. The results of this study show that *Baillonella toxisperma*, *Pentaclethra macrophylla* and *Trichoscypha abut* can considerably contribute towards the human nutrient requirements. These forest foods also contain substantial levels of health promoting phytochemicals notably flavonoids, polyphenols, vitamins C and E. These foods therefore have potential to promote nutrition and health, especially among forest dependent communities who consume them in substantial amounts.

## Background

Forest foods are a major source of food crops, providing significant amounts of nutrients and energy for millions of people in Cameroon, Gabon, Central African Republic, Congo Republic and DR Congo (Pimentel et al. 1997; Tieguhong et al. 2012). They also serve as a source of income for millions and have played a significant role in the economic growth and development of some Congo basin countries particularly Cameroon (Tieguhong et al. 2012). In Cameroon, the economic growth attributable to the forest foods is estimated to range between 6 and 10% per annum (Sonwa et al. 2012). Most forest foods are seasonal often harvested once or twice a year. Seasonal forest foods are sometimes processed and stored to ensure a year-round food supply, supplementing household diets and providing necessary nutrients during periods of food shortage (Sheil and Wunder 2002). Furthermore, as a result of the economic potential of some forest foods, some have been domesticated, forming an important component of the household subsistence cropping system that provides food to the family throughout the year (Ingram 2014).

Malnutrition is a serious problem among the forest dependent communities of Cameroon (Cameroon Demographic and Health Survey (CDHS) 2011). It is estimated that 60% of children aged 6–59 months in Cameroon are anemic, with children in rural areas having a higher anemia prevalence of 63% compared with children in urban areas who are 57% anemic. According to the World Health Organization, anemia is a major public health problem among children and adults in sub Saharan African countries including Cameroon (World Health Organization (WHO) 2008). It can lead to impaired cognitive development and performance, behavioral and language development. It has also been associated with low educational achievement as well as increased morbidity from infectious diseases (Saxton et al. 2009). At the same time, obesity and associated chronic diseases such as, hypertension and type I and type II diabetes are also a growing problem for Cameroon (Dongmo et al. 2007). There is increasing evidence of the role of plant based bioactive compounds in protection against chronic diseases (Thompson 1993; World Cancer Research Fund 1997). It has been shown that the bioactive compounds in forest and wild plant foods majorly consumed by the cattle keeping indigenous communities of the sub Saharan Africa play a considerable role in alleviating the potential health effects of the meat, milk and blood rich traditional diets of these communities (Johns et al. 1999). Inspite of the health benefits of traditional foods, imported rice and other foreign foods are increasingly replacing the locally available traditional and indigenous foods (FAO, WFP and IFAD 2012).

On the other hand, the report on the state of food insecurity describes a gloomy picture about the food and nutrition situation in Cameroon (FAO, WFP and IFAD 2012). It is estimated that if measures are not taken to reduce malnutrition, a 10% loss of lifetime earnings of an individual and 3% reduction in gross domestic product (GDP) are incurred in Cameroon and other tropical countries of Africa (World Bank 2006). Promotion of consumption of culturally acceptable forest sourced plant foods is a sustainable way of ensuring food and nutrition security (Pinstrup-Andersen 2009). This has potential to complement existing interventions and to offer a sustainable and low cost way to reach vulnerable populations (Ouedraogo et al. 2009). In addition to the likely economic benefits, advantages of such interventions include empowerment of individuals and households, leading to wise food selection, family food production and provision of multiple nutrients simultaneously (Ruel 2001) and an enhancement of cultural pride and identity (Oniang'o et al. 2005). However, there is paucity of data on the proximate composition and bioactive contents of forest plant foods in Cameroon. Previous data on analysis of Cameroonian forest plant foods for nutrient and bioactive compounds are limited to nutrient composition data for foods consumed in northern part of the country (Djoulde et al. 2012) and phytochemical constituents of five selected medicinal plants (Dongmo et al. 2007). Forest foods composition data are needed to identify forest plant foods that can be promoted and to provide basis for advocacy to promote consumption of these foods. Therefore, the purpose of this study was to investigate nutritional value, bioactive and anti-nutritional composition of forest plant foods with high cultural acceptability and potential for increasing nutrient status in the population.

## Materials and Methods

### Sampling and sample preparation

**Figure 1:**
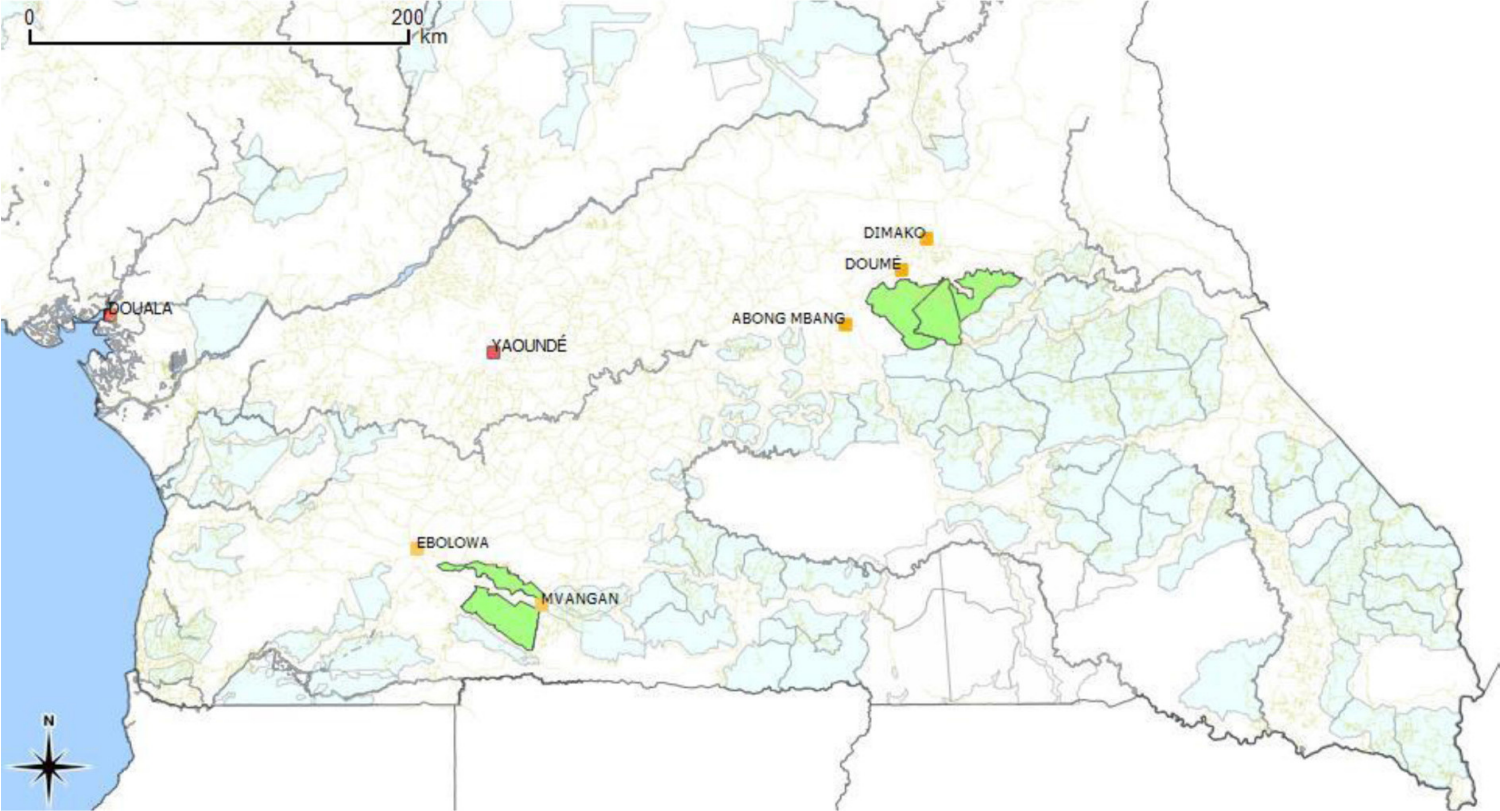
Location of study sites.

**Figure 2:**
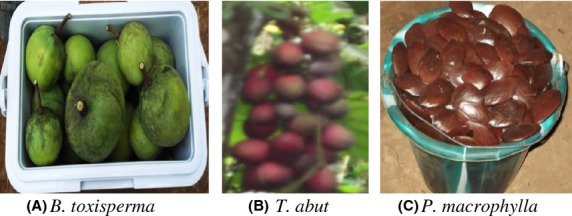
Edible portions of *Baillonella toxisperma*, *Trichoscypha abut* and the nut of *Pentaclethra macrophylla*.

Three readily available and widely consumed edible parts of forest food species including; *Baillonella toxisperma* (Moabi), *Trichoscypha abut* (Mvout) and *Pentaclethra macrophylla* (Ebaye) were sampled from the east and south sites (Fig.1). The three are timber tree producing species and consist of two fruits of *B. toxisperma* and *T. abut* and the nuts of *P. macrophylla*. Three villages were selected from each site, on the basis of their accessibility, ethnicity and proximity to the annually allocated timber logging areas (Fig.2). The population around the eastern site is numbered about 25,783 people who live in 41 villages and are mainly of the Kako, Pol, Maka and Baka pygmy ethnic groups (Medinof 2004). The population in the south region site is estimated at 79,353, living in 29 villages (Enviro Consulting 2009), nearly all of the Bulu ethnic group. The study villages were stratified according to ethnicity and the level of forest exploitation by the logging companies. A multi-stage cluster sampling technique involving one stage of purposeful selection and one stage of randomization was deployed. In the first stage the most accessible administrative districts within each site and fitting the village selection criterion listed above were purposefully selected. In the second stage, three villages were randomly selected from the selected districts of each site. From the east, samples were collected from the villages of Melabo, Nkolbikong and Bonando while in the south samples were collected from the villages of Ngong, Bissam and Ondondo.

From each study village, an average of 5 mature fresh fruits and nuts per species, were sampled from different points and collected in a perforated plastic container, labeled and kept in an ice box container and transported to the laboratory at Yaoundé I University in Cameroon for analyses. At the laboratory, the samples were washed thoroughly with deionized water and conserved in a refrigerator at 4°C. For each fruit species, two fruits out of the three per village were randomly selected for edible pulp extraction. The pulp from the fruits and nuts were mashed in a blender. Extracted edible pulp per species were divided into two sub samples. For the first sub sample, the extracted fresh pulp was immediately sealed in clean polyethylene bags and conserved at −20°C and later used for vitamin, bioactive compound and anti-nutrient analysis, while the other sub sample was analyzed for moisture content. The dried samples were conserved for proximate analysis.

### Fruit nutrient analyses

All reagents, reference standards and organic solvents were purchased from Merck (Darmstadt, Germany).

#### Proximate composition

Proximate composition was determined using the AOAC methods (AOAC 2006). The moisture content was determined using the vacuum oven method 934.01, crude oil content by the ether extraction method 920.39, total ash by method 942.05, crude fibre by method 978.10, crude protein by the Kjeldahl 984.13 method, total carbohydrates by difference: 100% − (crude protein% + ash% + crude fat % + moisture%) and metabolizable carbohydrates by difference calculation: 100%− % (crude protein% + ash% + crude fat% + moisture% + crude fiber%).

#### Minerals

Samples for determination of mineral content were digested using nitric/sulphuric acid (1:1 v/v) mixtures (AOAC 2006). The mineral constituents (calcium [Ca], copper [Cu], magnesium [Mg], and zinc [Zn] were determined by the atomic absorption spectrophotometric method 975.03B (b) (AOAC 2006). Iron was determined using the method 999.11 (AOAC 2006). Selenium [Se] was determined using the method 996.16(G) (AOAC 2006). Potassium [K] and sodium [Na] were separately determined calorimetrically, using the flame emission photometry method 956.01 (AOAC 2006). Phosphorous [P] was determined using the gravimetric method 966.01 (AOAC 2006). The determined mineral concentrations of each sample were quantified by comparison, against the standard curves. Standard curves were obtained after calibrations were performed using external standards for each corresponding pure mineral of sodium, magnesium, iron, zinc, selenium, potassium and calcium and phosphorous and prepared from a 1000 ppm single stock solution made up with 2% nitric acid. The external calibrations were run in the same analytical sequence as the samples (AOAC 2006).

### Bioactive compounds analysis

#### Flavonoids, polyphenols, proanthocyanins, carotenoids, vitamin C and E, phytic acid, total oxalates and tannins

Chemical analysis for bioactive compounds was done on extracts of fresh fruits (20 g) from each study sample. For flavonoids and polyphenols extraction, the pulp of the fruits was carefully removed from seed. A blender (Magic Line, Model MFP 000, Nu World Ind. (Pty) Ltd, Johannesburg, South Africa) with stainless steel blades was used to grate and blend the pulp to fine pulp. The fine pulp was stirred continuously with 50 mL (80% v/v) methanol for 24 h. The extract was filtered and the filtrate was centrifuged at 4000 rpm Xg for 15 min using a centrifuge (Hitachi Model CR22N CE; Hitachi Koki Co., Ltd Life sciences instruments, Tokyo, Japan). The supernatant was stored at 4°C prior to use within two days. Total phenolic content in the methanolic extract was determined by Folin-Ciocalteu's calorimetric method (Amin et al. 2004). The absorbance was measured at 765 nm using UV-VIS spectrophotometer (U-2001; Hitachi Instruments Inc., Tokyo, Japan). Quantification was done on the basis of standard curve of gallic acid prepared in 80% methanol (v/v) and results were expressed in milligrams gallic acid equivalent (GAE) per gram fresh weight (fw) of fruits (Georgé et al. 2005). Total flavonoid content in the methanolic extract of plant samples was determined by aluminum chloride calorimetric method (Zhishen et al. 1999). The absorbance was measured at 415 nm UV-VIS spectrophotometer (U-2001; Hitachi Instruments Inc., Tokyo, Japan). Quantification of flavonoids was done on the basis of standard curve of quercetin prepared in 80% methanol and results were expressed in milligram quercetin equivalent (QE) per gram fruit weight. Proanthocyanins were extracted from selected samples using 70% (v/v) ethanol in water, overnight at room temperature. The total content of proanthocyanins in fruit extracts was determined spectrophotometrically by the pH differential method (Giusti et al. 1999). The UV-Visible spectrophotometer (U-2001; Hitachi Instruments Inc., Tokyo, Japan) was used to read absorbance at 530 and 700 nm. The total proanthocyanins content was calculated according to the standard curve of pure cyanidin-3-O-glucoside (Darmstadt, Germany) and expressed as cyanidin-3-O-glucoside (Cyn-3-O-G) mg/100 gs fresh weight. Vitamin C (ascorbic acid) was analyzed by spectrophotometric measurements at 515 nm against a blank, after being extracted using Phosphotungstate reagent (PR) (Vanderslice and Higgs 1990). The content of vitamin C was calculated on the basis of the calibration curve of authentic L-ascorbic acid that acted as the standard reference.

*β*-carotene content was determined colorimetrically using AOAC method No 970.64 (AOAC 2005) after extraction with xylene and separation by column chromatography. *β*-carotene was determined by measuring absorbance at 470 nm against a blank sample. Standard curves were made with pure *β*-carotene standard and the results ex- pressed as mg *β*-carotene. Vitamin E (tocopherol*)* was extracted using alcoholic sulphuric acid and absolute alcohol method AOAC 971.30 method (AOAC 2005) and the concentration was calculated from the absorbance measured by a Spectrophotometer (UV/VISIBLE). Vitamin E was determined by measuring absorbance at 270 nm against a blank sample. Standard curves made with pure tocopherol were used for this purpose and the results expressed as mg vitamin E equivalent per 100 g.

The anti-nutrients phytic acid, total oxalates and tannins were determined from different extracts of the samples. Total oxalates were determined by extraction of samples with hydrochloric acid and soluble oxalate. The total oxalate concentration in the fruits were estimated using the spectrophotometric method (U-2001; Hitachi Instruments Inc., Tokyo, Japan), by reading the extracts absorbance and comparing it with the absorbance of the authentic calcium oxalate at 420 nm (Jones John 1988). Tannins were determined by extracting the fruit pulps with methanol and measuring absorbance at 500 nm (Griffiths and Jones 1997). Phytic acid was extracted and determined according to the precipitate analysis method of Thompson and Erdman (Thompson and Erdman 1982). The conversion factor 3.55 for phosphorus to phytic acid was used. Pure phytic acid was used as a standard.

All the analyses were conducted in triplicate.

### Data analysis

All of the statistical analyses were carried out using statistical software SPSS version 21, with results being expressed as means ± standard deviations of three separate determinations. Using the estimated total daily food intake estimations of forest communities in Cameroon (Yamauchi et al. 2000), including; 200 g for a child aged 1–3 years and 300 g for a non-lactating non-pregnant woman, the possible potential impact of the forest foods on the daily nutrients requirements among children and adults was calculated. Calculations were done to show the potential contribution of forest foods to daily recommended dietary allowances (RDA) of either the children or the adults.

## Results

### Proximate composition

*Baillonella toxisperma* and *Trichoscypha abut* were found to be high in carbohydrates, with total content exceeding 88% (Table1). *P. marcrophylla* on the other hand was found to contain substantial levels of fat, protein and dietary fiber.

**Table 1:** Mean (% edible portion wet basis) and standard deviations for proximate composition of *Baillonella toxisperma, Trichoscypha abut and Pentaclethra macrophylla*^1^

Proximate Composition^2^	Mean concentrations (%)		
	B. toxisperma	P. macrophylla	T. abut
Water content	74.20 ± 2.32	44.85 ± 19.80	85.96 ± 0.67
Total fat	9.25 ± 2.27	38.71 ± 6.72	11.04 ± 0.10
Ash	0.58 ± 0.47	1.21 ± 0.72	4.70 ± 0.31
Total protein	1.15 ± 2.10	15.82 ± 3.12	0.52 ± 0.6
Total carbohydrates	89.59 ± 2.33	45.47 ± 7.70	88.45 ± 0.9
Digestible Carbohydrates	84.18 ± 2.42	28.37 ± 8.51	81.41 ± 0.96
Total dietary fiber	5.41 ± 0.94	17.10 ± 2.82	7.04 ± 0.89

1 Each value is the mean and standard deviation of 9 sample lots analyzed individually.

2 Edible portion wet basis.

### Minerals content

Overall the nuts of *P. macrophylla* contained exceptionally high content of several minerals including; sodium, magnesium, iron, zinc and selenium (Table2). Potassium and calcium were highest in the fruits of *B. toxisperma*. Although phosphorous was considerably high in the fruits of *T. abut*, overall the fruits of *T. abut* contained remarkably low mineral contents.

**Table 2:** Mean concentrations (mg or *μ*/100 g edible portion wet basis) and standard deviations of minerals of *Baillonella toxisperma*, *Trichoscypha abut* and *Pentaclethra macrophylla*^1^

Macro and micro minerals^2^	Mean concentrations (mg/100 g)		
	B. toxisperma	P. macrophylla	T. abut
Na	9.5 ± 1.8	15.4 ± 1.5	0.6 ± 0.1
K	27.5 ± 5.6	10.9 ± 1.8	8.2 ± 0.1
Ca	37.5 ± 5.2	36.6 ± 8.7	33.8 ± 4.03
Mg	12.3 ± 6.3	36.3 ± 5.2	11.7 ± 0.9
P	11.9 ± 3.2	30.8 ± 6.1	45.1 ± 1.2
Fe	3.3 ± 1.3	5.4 ± 0.5	1.2 ± 0.2
Zn^3^	0.2 ± 0.1	1.1 ± 0.2	0.1 ± 0.02
Se^3^	0.1 ± 0.02	0.2 ± 0.02	0.001 ± 0.0002

1 Each value is the mean and standard deviation of 9 sample lots analyzed individually.

2 Edible portion wet basis.

3 *μ*/100 g.

In each raw different letters mean significant differences of averages (*P* < 0.05).

### Bioactive compounds

#### Flavonoids, polyphenols, proanthocyanins, carotenoids, vitamin C and E, phytic acid, total oxalates and tannins

The fruits of *T. abut* generally exhibited high content of bioactive compounds including flavonoids, polyphenols, vitamin C, total oxalates and proanthocyanins (Table3). *β*-carotene and vitamin E were considerably high in the fruits of *B. toxisperma*. The highest Vitamin E content in the present study registered in nuts of *P. macrophylla* was two folds higher than the content registered in the fruits of *B. toxisperma* and 1200 folds higher than the content registered in fruits of *T. abut*. Overall, the nuts of *P. macrophylla* contained considerable content of bioactive compounds with anti-nutritional properties. These include; phytic acid and tannins. However, tannins also have positive bioactive properties.

**Table 3:** Mean concentrations (mg or *μ* or g/100 g edible portion wet basis) and standard deviation of bioactive compounds in *Baillonella toxisperma*, *Trichoscypha abut* and *Pentaclethra macrophylla*^1^

Bioactive compounds^2^	Mean concentration ±SD (mg/100 g)		
	B. toxisperma	P. macro-phylla	T. abut
Flavonoids	141.1 ± 15.2	146.1 ± 59.2	306.0 ± 15.0
Polyphenols	686.7 ± 89.6	671.8 ± 147.1	947.0 ± 15.5
Proanthocyanins	28.0 ± 8.4	65.0 ± 29.1	61.2 ± 83.03
Vitamin C	50.3 ± 7.7	9.5 ± 0.9	80.1 ± 1.8
Carotenoids (*β*-carotene)^3^	17.9 ± 2.7	6.8 ± 1.6	0.9 ± 0.1
9-vitamin E	9.3 ± 1.6	19.4 ± 4.3	0.02 ± 0.001
Phytic acid^3^	0.2 ± 0.02	1.8 ± 0.21	0.1 ± 0.0002
Total oxalates^4^	0.01 ± 0.001	0.2 ± 0.03	0.6 ± 0.002
Tannins^4^	0.2 ± 0.008	0.4 ± 0.4	0.003 ± 0.001

1 Each value is the mean and standard deviation of 9 sample lots analyzed individually.

2 Fresh weight basis.

3 *μ*/100 g.

4 g/100 g.

In each raw different letters mean significant differences of averages (*P* < 0.05).

## Discussion

### Proximate composition

The lipids, protein and dietary fiber contents were remarkably high in the nuts of *P. macrophylla*. Previous findings in *P. macrophylla* from Nigeria, revealed a lipid content of 47.9% (Ikhuoria et al. 2008) and fiber content ranging from 19.0% (Akindahunsi 2004) to 21.7% (Ikhuoria et al. 2008). The difference between the contents from the three studies may possibly be due to differences in growth conditions, genetic variation, or probably due to differences in post-harvest handling, processing, storage conditions and stage of maturity (Rodriguez-Amaya and Kimura 2004). Compared to commonly consumed oil producing foods, the lipid content of *P. macrophylla* in the present study is higher than the value of 23.5% reported in soybeans (*Glycine max*) (Olaofe et al. 2006), one of the most widely consumed oilseeds globally. Based on its’ high fat content, the nuts of *P. macrophylla* can be considered a good source of dietary energy and could be considered for use in production of high energy foods such as complementary foods and foods for emergency situations. Soybeans are the main ingredient used in production of such foods. Furthermore, the highest protein content, in the nuts of *P. macrophylla* of the present study, was higher than the content of 9.31% reported by Ikhuoria et al. (2008), but lower than the content of 20.94% reported by Alinnor and Oze (2011), in the Nigerian grown nuts of *P. macrophylla*. However, the overall protein content of the three forest foods in the present study, were less than the content reported in other commonly consumed exotic plant nuts in Cameroon, including Bambara groundnut (*Vigna subterranean*) that have a protein content of 20.6% (Mazahib et al. 2013) and soybeans which have an average protein content of 45% (Cheftel et al. 1985).

The fruits of *B. toxisperma* and *T. abut* forest foods were found to contain substantial amounts of carbohydrates. The carbohydrate content in the two forest foods were higher than some commonly consumed forest fruits such as bush mangoes (*Irvingia gabonensis)* (7.4–13.5%), *Ricinodendon heudolitii* (0.8–5.6%) and *Dacryodes edulis* (4.5–8.7%) as reported by Vincenti et al. (2008). However, the protein and fat content of the fruits of *B. toxisperma* and *T. abut* forest foods are considerably lower than the contents reported in the fruits of *I. gabonensis, R. heudolitii* and *D. edulis* (Vincenti et al. 2008).

### Minerals concentration

The mineral content in the three forest foods in this study were in the range or higher than mineral contents reported in previous studies of nuts of *P. macrophylla* (Akindahunsi 2004; Enujiugha and Akanbi 2005; Ikhuoria et al. 2008) and commonly consumed forest fruits; *I. gabonensis*, *R. heudolitii* and *D. eduli (*Vincenti et al. 2008). The essential minerals of iron and zinc in the nuts of *P. macrophylla*, were within the range of the previous findings of the same species. A range of 0.98–1.8 mg/100 g for zinc (Akindahunsi 2004; Enujiugha and Akanbi 2005) and a range of 1.7–5.6 mg/100 g for iron (Enujiugha and Akanbi 2005; Ikhuoria et al. 2008) was reported in the *P. macrophylla* nuts grown in Nigerian forests. Also, the sodium, calcium, and magnesium contents in *P. macrophylla* of the present study, were higher than contents of the same minerals reported previously in Nigerian *P. macrophylla* (Akindahunsi 2004; Enujiugha and Akanbi 2005; Ikhuoria et al. 2008). The difference between the contents from the four studies may be attributable to genetic variation, or probably due to differences in post-harvest handling and stage of maturity (Rodriguez-Amaya and Kimura 2004). Sodium content of 10.2 mg/100 g and magnesium content of 9.7 mg/100 g were reported by Akindahunsi (2004) while Ikhuoria et al. (2008) reported calcium content of 8.2 mg/100 g.

Whereas the mineral contents in the fruits of *B. toxisperma* and *T. abut* fall in the range of mineral contents of the commonly consumed forest fruits of *I. gabonensis,* they were generally lower than the contents reported in the fruits of *D. eduli* and *Canarium schweinfurthii* (Mbelli, 2002; Vincenti et al. 2008). For example the calcium, iron and zinc contents registered in the two fruits of *B. toxisperma* and *T. abut* are comparable with calcium (23.2 mg/100 g), iron (4.7 mg/100 g) and zinc (1.2 mg/100 g) contents in the forest fruits of *I. gabonensis* (Vincenti et al. 2008). However the calcium, iron, magnesium and zinc contents in the two forest fruits are lower than contents reported in *D. eduli* (Vincenti et al. 2008) and *C. schweinfurthii* (Mbelli, 2002).

In comparison, to the estimated FAO/WHO recommended daily intake (RDA) (FAO/WHO 1996) and the estimated daily portion intake for adults and children in Cameroon (Yamauchi et al. 2000), the three forest foods can have a substantial contribution to the requirements (Table4). Analysis of the nutrient content of the forest foods against RDA showed that a portion of 200 g of the fruits of *B. toxisperma* and the nuts of *P. macrophylla* can supply 15–95%, 10–50% and 20%, respectively of iron, zinc and calcium requirements for a child aged between 1 and 3 years. It also revealed that fruits of *B. toxisperma* and *T. abut* can supply more than 70%, of the total magnesium requirement of 60 mg/day among children aged 1–3 years and that the nuts of *P. macrophylla* supply 100% total magnesium requirements to children. Iron and zinc deficiencies are serious problems affecting millions of children in Cameroon and the sub Saharan Africa region (Pinstrup-Andersen 1997). Similarly, 300 g of *P. macrophylla* nuts meets approximately 50% of the recommended daily requirements of 220 mg for magnesium, 30% total daily iron and zinc requirements of 58.8 mg and 12 mg respectively, for a non-pregnant non lactating woman. Of the seven minerals studied, the three forest foods were found to have adequate levels to significantly contribute to the RDA requirements for five, namely, iron, zinc, selenium, magnesium and calcium.

**Table 4:** Contribution of consumption of the Cameroonian forest plant foods (200 gs among children and 300 gs among adults) to the daily requirements of major and minor nutrients

Micro nutrients	Children (1–3 years) (mg/day)			Adults (19–60 years) (mg/day)			RDA children (mg/day)	RDA adults mg/day
	Baillonella toxisperma	Pentaclethra macrophylla	Trichoscypha abut	Baillonella toxisperma	Pentaclethra macrophylla	Trichoscypha abut		
Na	19.0	30.7	1.16	28.6	46.1	1.7	400	500
K	55.0	21.7	16.3	82.6	32.7	24.5	1600	2000
Ca	75.0	73.2	67.5	112.6	109.6	101.3	500	1000
Mg	24.6	72.6	23.5	35.7	108.9	35.2	60	220
Fe	6.6	10.9	2.3	9.93	16.3	3.5	11.6	58.8
Zn^1^	0.5	2.3	0.2	0.7	3.39	0.3	4.5	12
Selenium^1^	0.1	0.4	0.003	0.12	0.54	0.004	13.6	20.4
Vitamin A RE^2^	5.9	2.27	0.3	8.9	3.4	0.5	400	500
Vitamin C	100.6	18.9	160.1	150.8	28.4	240.2	30	45
Vitamin E	18.6	38.8	0.03	27.9	58.2	0.1	0.3	0.4

1 Units of measurement are *μ*/100 g.

2 Retinol equivalents (REs) (conversion factor 6:1 from *β*-carotene equivalents to RE)

Source: (37)

### Bioactive compounds

#### Flavonoids, polyphenols, proanthocyanins, carotenoids, vitamin C and E, phytic acid, total oxalates and tannins

Overall the fruits of *T. abut* contained considerably high contents of polyphenols, flavonoids and vitamins C and E, than the values reported for some forest foods and commonly consumed plant foods in Cameroon and other countries. Vitamin C content of the fruits of *T. abut* is more than, eight folds the content reported in the forest fruits of *R. heudolitii* (7.5 mg/100 g) and *Tamarindus indica* (9 mg/100 g), more than two folds the content in *D. eduli* (32.1 mg/100 g) and slightly higher than the content in *I. gabonensis* (66.4 mg/100 g) and *Sclerocarya birrea* Hochst (68 mg/100 g) (Ejiofor et al. 1987; Vincenti et al. 2008; Kehlenbeck et al. 2013). *T. abut* vitamin C content recorded in this study is about seven folds higher than the content reported in dessert bananas and two folds higher than that of *papaya* (51.2 mg/100 g) (Marisa 2006). Based on the FAO/WHO recommended daily intake (RDA) (FAO/WHO 1996), approximately 200 g and 300 g of fruits of either *B. toxisperma* or *T. abut* are required to supply the recommended daily intake for vitamin C of 30 mg and 45 mg for children and adults, respectively (Table4). The highest flavonoid content in the fruits of *T. abut* was remarkably higher than the flavonoid content in some popular wild forest foods in west and central Africa (Lamien-Meda et al. 2008). Flavonoid content of fruits of *T. abut*, is 30, 11, 10 and 7 times the values reported in forest fruits of *Dialium guineense* (10.23 mg/100 g), *Diospyros mespiliformis* (27.10 mg/100 g), *Vitellaria paradoxa* (30.95 mg/100 g) and *Adansonia digitata* (42.73 mg/100 g), respectively.

The nuts of *P. macrophylla* contained the highest proathocyanins and vitamin E contents. The vitamin E content recorded in the nuts of *P. macrophylla* were considerably higher than the contents reported in some of the forest nuts of *Parkia biglobosa* (18.13 mg/100 g) (Olujobi 2012). The results also revealed that approximately 300 g of either the fruits of *B. toxisperma* or the nuts of *P. macrophylla* would be sufficient to supply one-third of the daily vitamin E requirement of 0.4 mg and 0.3 mg respectively for non-pregnant non-lactating female and for children aged 1–3 years. These results show that the three forest food plants analyzed in this study could play a considerable role in meeting the dietary vitamin C and vitamin E requirement for forest dependent communities in Cameroon. Overall the phenolic content was high in all the studied fruits and the nut samples as compared to exotic fruits (Hukkanen et al. 2006; Lako et al. 2007; Lim et al. 2007). The lowest polyphenol contents in the nuts of *P. macrophylla* were as high as two to three folds that in regularly consumed exotic fruits of blueberries (670.9 mg/100 g), dog berries (432 mg/100 g) and sour cherries (429.5 mg/100 g) (Proteggente et al. 2002; Marinova et al. 2005; Hukkanen et al. 2006; Lim et al. 2007). The phytic acid, tannins and oxalates content of the nuts of *P. macrophylla*s in the present study are similar to values of 2.11 g/100 g for phytic acid, 0.38 g/100 g for tannins and 2.79 g/100 g for oxalates previously reported for nuts of *P. macrophylla*s grown in Nigeria (Akindahunsi 2004). High content of flavonoids, phenols and proanthocyanins is associated with high antioxidant activity and the prevention of cell destruction and other diseases mediated by oxidative stress (Vinson et al. 1995a,b1995a; Hollman et al. 1996; Floegel et al. 2011). Flavonoids, phenols and proanthocyanins have also been shown to control diarrhea and diabetes (Vinson et al. 1995a,b; Favier 2003; Agbor et al. 2004). For example the nuts and leaves of *P. macrophylla* have been used to treat gonorrhea among forest dependent communities of Cameroon (Ndenecho 2009), while *B. toxisperma* has been used to treat rheumatism and child birth shocks among women in Cameroon (Jiofack et al. 2010). *P. Macrophylla* has also previously been reported to exhibit the antimalarial and anti-diabetic effects and this is attributed to their content of a wide range of antioxidant components (Food and Agriculture Organization (FAO) 2001).

The highest *β*-carotenoid content for the foods studied was registered in the fruits of *B. toxisperma*. The recorded *β*-carotenoid content of *B. toxisperma* (17.9 *μ*/100 g) was however, remarkably lower than the values reported in commonly consumed *β*-carotene rich foods such as *papaya* (232.3 *μ*/100 g), desert bananas (96.9 *μ*/100 g) and cooking bananas (337 *μ*/100 g) (Marisa 2006; Fungo et al. 2010). The forest foods in the present study can therefore not be considered as good dietary sources of pro-vitamin A.

## Conclusions

The findings of this study indicate that the three foods that were investigated were nutritionally diverse. Of the three foods, *T. abut* exhibited considerably high content of bioactive compounds including flavonoids, polyphenols, proanthocyanins and vitamin C. *P. macrophylla* nuts had the highest content of iron, zinc, magnesium, calcium and vitamin E. Based on their nutritional value, it can be concluded that the three foods can make considerable contributions towards meeting nutrient requirements, for iron, zinc, vitamins C and E. The forest foods are also good sources of health promoting phytochemicals. There is need to disseminate information about the nutritional and phytochemical composition of these foods to promote their consumption.
